# The global poliovirus eradication initiatives in Kano state, Nigeria: a case report on the African regional commission pre-certification visit and lessons learnt

**DOI:** 10.3389/fpubh.2025.1690423

**Published:** 2025-10-27

**Authors:** Eric Okorie Nwaze, Igbaver Isaac Ieren, Chinenye Okafor

**Affiliations:** ^1^Department of Community Medicine, David Umahi Federal University of Health Sciences, Uburu, Nigeria; ^2^National Primary Healthcare Development Agency (NPHCDA), South East Zone, Enugu, Nigeria; ^3^Saskatchewan Ministry of Health, Regina, SK, Canada; ^4^World Health Organization Health Workforce Department, Geneva, Switzerland

**Keywords:** Kano, Poliovirus, Nigeria, pre-certification, eradication

## Abstract

**Background:**

Polio eradication in Kano State, Nigeria, represents a major milestone in the Global Polio Eradication Initiative (GPEI). Formerly the epicentre of wild poliovirus (WPV) in Africa, Kano experienced multiple outbreaks between 2003 and 2008, threatening national and regional stability. Persistent transmission, vaccine resistance, and surveillance gaps kept Kano in global focus. By 2020, following intensive interventions, Kano was certified polio-free by the African Regional Certification Commission (ARCC).

**Methods:**

This narrative report draws from ARCC field verification visits, peer-reviewed literature, unpublished reports from the National Primary Health Care Development Agency (NPHCDA), and records from WHO, UNICEF, and partners. Data (2008–2020) included surveillance indicators, immunisation coverage, cold chain assessments, supplementary immunisation activities (SIAs), and stakeholder interviews. Emphasis was on Acute Flaccid Paralysis (AFP) surveillance, technological innovations such as AVADAR and GIS mapping, and the role of traditional and religious leaders in overcoming resistance.

**Results:**

Kano achieved AFP surveillance sensitivity above the WHO benchmark (2/100,000 children under 15), expanded environmental surveillance, and improved routine immunisation with coverage exceeding 80% in most Local Government Areas by 2019. ARCC verification noted strong documentation, political commitment, advocacy, and correction of case investigation and outbreak records.

**Conclusion:**

Kano’s transformation from a WPV hotspot to polio-free status resulted from integrated strategies combining technology, advocacy, surveillance, and independent verification. These lessons offer a model for sustaining polio-free gains, addressing circulating vaccine-derived polioviruses, and strengthening wider health systems.

## Background

Since 1988, the Global Polio Eradication Initiative (GPEI) has driven a > 99% reduction in polio incidence worldwide, yet the final mile has repeatedly tested the resilience of health systems. Nigeria—Africa’s last reservoir of WPV—was at the centre of global attention for much of the 2000s and early 2010s, Polio eradication in the country, particularly in Kano State, represents a compelling public health success story. Successes from small scale interventions piloted in Kano were later adopted for polio eradication efforts in other parts of the country and beyond Nigeria’s shores (1). Once the epicentre of wild poliovirus (WPV) transmission and a source of cross-border outbreaks, Kano’s reversal was achieved through a robust combination of strategies (2, 3). For this state its dense urban settlements, extensive peri-urban sprawl, and vibrant trade links formed efficient conduits for virus transmission. Equally consequential were the sociocultural dynamics that fomented vaccine hesitancy following the 2003–2004 Oral Polio Vaccine (OPV) controversies, when rumours of contamination and infertility risks circulated widely. As a result, routine coverage faltered, Supplemental Immunisation Activiteis (SIAs) encountered pockets of non-compliance, and chains of transmission extended beyond state and national borders.

As of June 2008, the burden of type 1 wild polio virus (WPV1) here was describe to be threatening neighbouring countries as over 90 cases were reported in the state with direct link to cases in countries with adjoining borders (4–8). The state had a known history of high WPV burden and was identified as a leading source of this crippling disease to other parts of the country and beyond (4). Wild polio virus transmission interruption eventually happened in 2015 through the National Primary Health Care Development Agency (NPHCDA) led collaboration with multiple stakeholders that included partner development organisations, religious, and traditional leaders (9).

NPHCDA, in concert with WHO, UNICEF, the Bill & Melinda Gates Foundation, Centre for Disease Control (CDC) (10), Rotary, and local civil society, implemented a layered portfolio: high-frequency SIAs, improved microplanning, independent monitoring, transit vaccination at borders and motor parks, and the progressive use of digital tools. Emergency Operations Centres (EOCs) provided daily incident-management routines and partner alignment. Meanwhile, engagement of traditional rulers and Muslim clerics reshaped the social narrative from scepticism to advocacy, especially as programme teams demonstrated transparency, responsiveness to concerns, and tangible co-benefits—such as integrated child-health services delivered alongside SIAs (11–13).

The ARCC, under the WHO Africa Regional Office, provided the framework and validation tools needed to confirm eradication (1). The commission set stringent documentation and field-verification requirements. Achieving certification readiness demanded that states not only interrupt transmission but also demonstrate surveillance sensitivity and high-quality documentation over time. The ARCC’s 2020 visit to Kano focused on validating Acute Flaccid Paralysis (AFP) and environmental surveillance performance, Routine Immunisation (RI) progress, SIA quality, cold-chain functionality, microplanning fidelity, and the ability of the system to detect and respond to potential importations or emergent circulating vaccine-derived polioviruses (cVDPVs).

This narrative report covered the visit of the ARCC to Kano State which is one of the 37 states including the Federal Capital in Nigeria. The state is located in the North Western region of the country. It has 44 Local Government Areas of varying diversity, which is the highest in Nigeria.

### Objectives

1. To reconstruct the ARCC verification pathway in Kano and summarise the programmatic evidence presented.
2. To analyse the enabling determinants—governance, technology, social mobilisation, and systems discipline—that shifted Kano from a high-risk to a certified setting.
3. To extract lessons to sustain polio-free status and to inform analogous campaigns (e.g., measles, yellow fever) and primary health-care strengthening in Nigeria and comparable contexts.

## Methods

**Figure 1:**
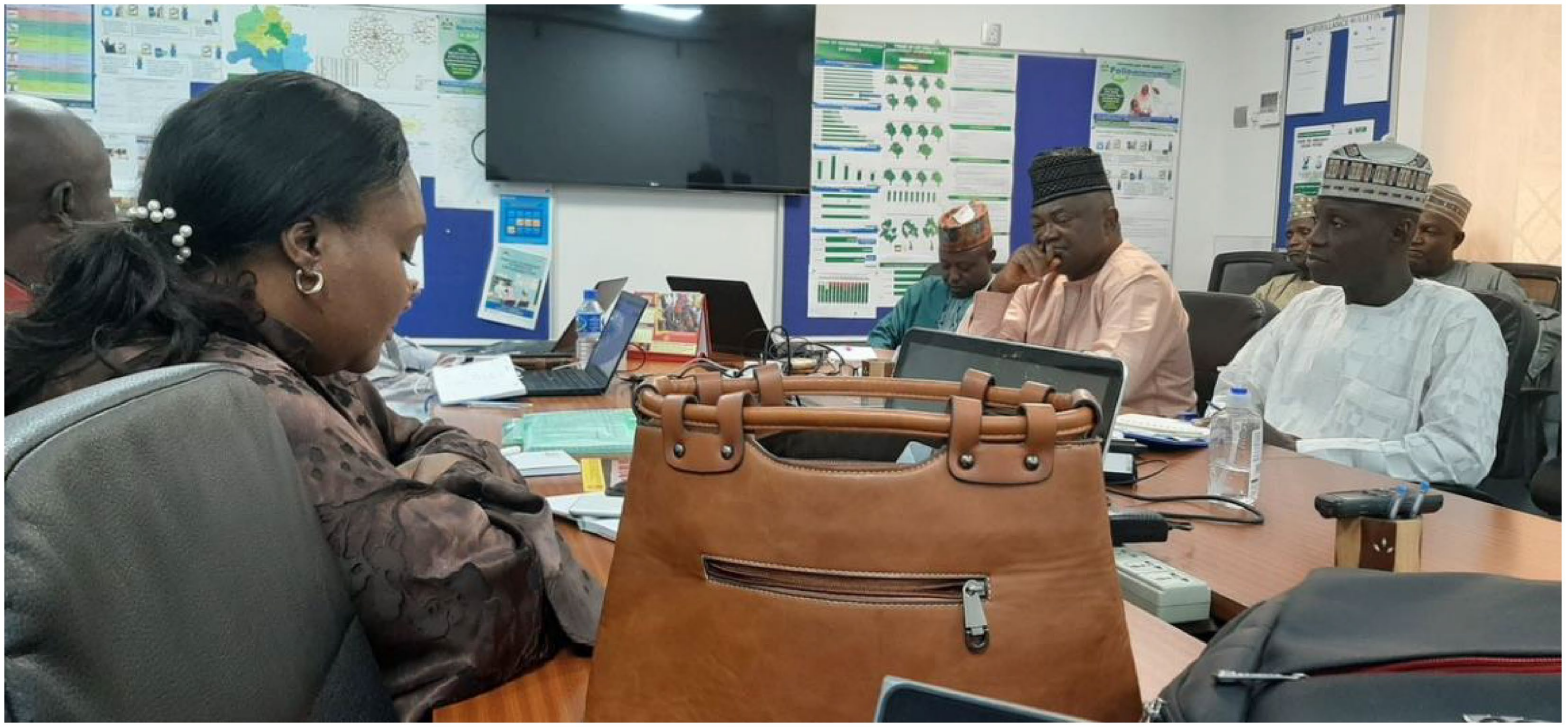
ARCC team reviewing documents and brainstorming in Kano.

**Figure 2:**
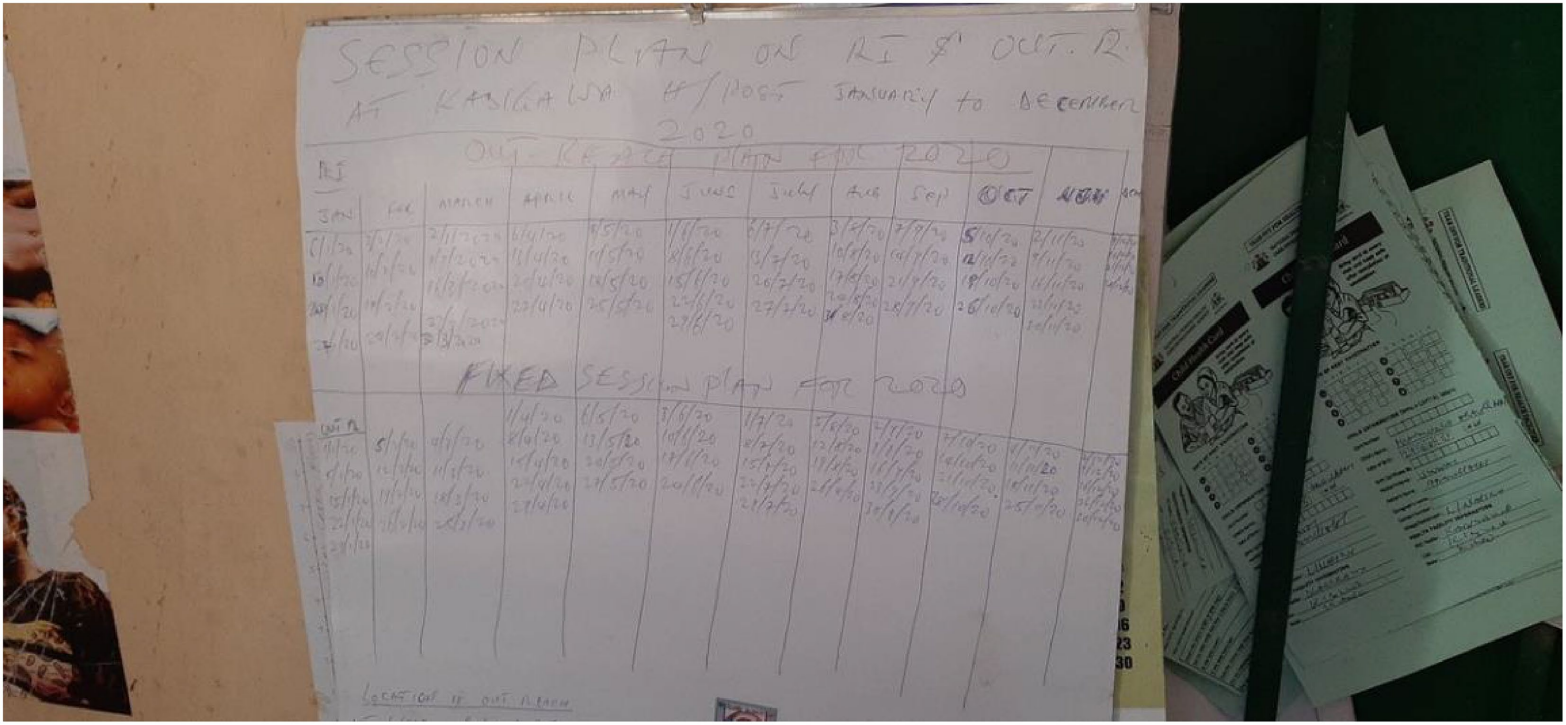
Health facility service records undergoing data extraction during ARCC Visit in Kano.

**Figure 3:**
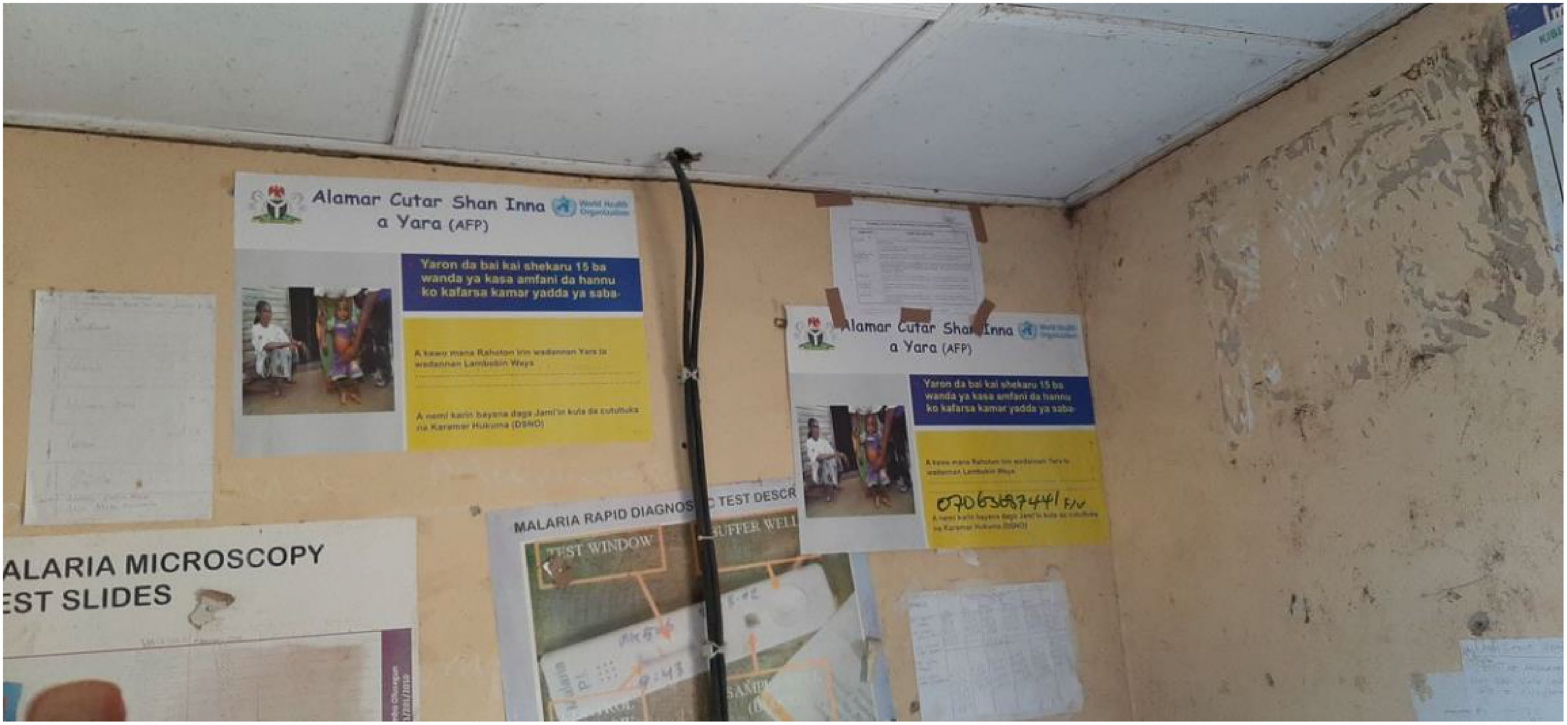
Pictorials were visibly displayed in one health facility at Kibiya LGA.

**Figure 4:**
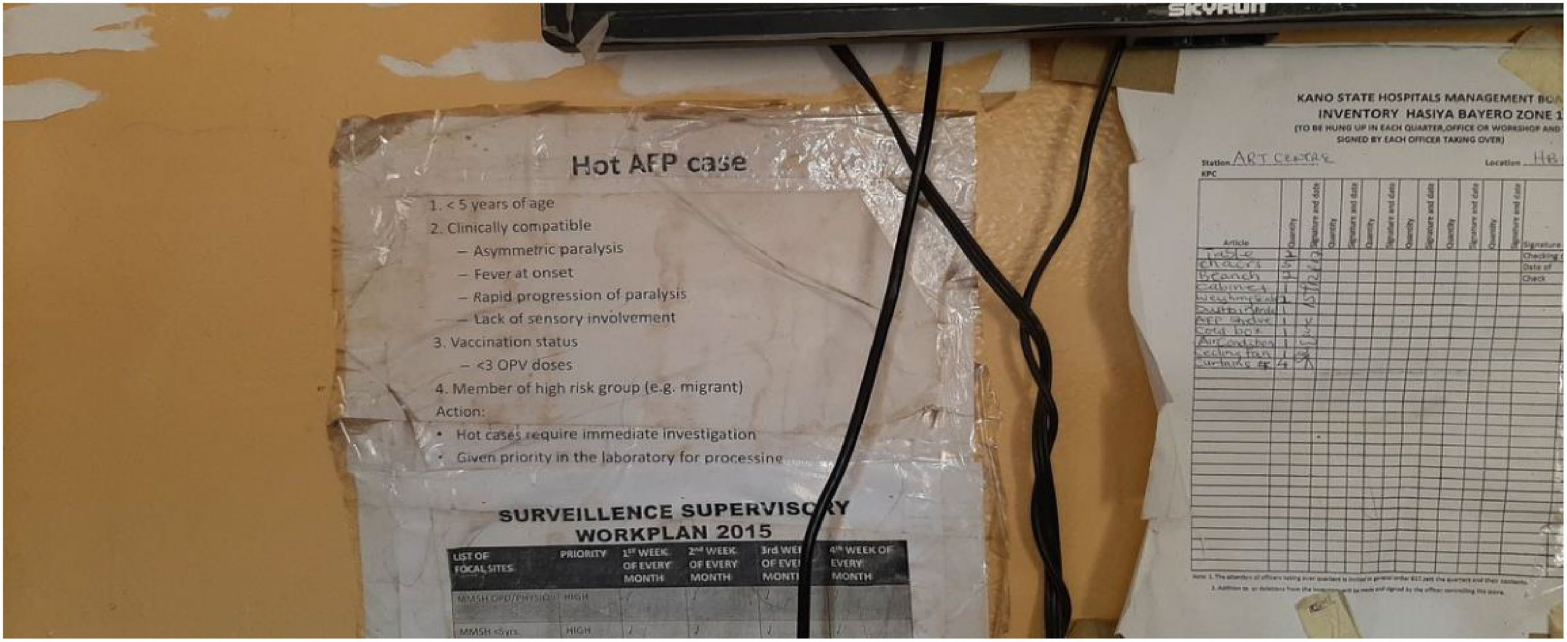
AFP and immunization records were reviewed and data extracted during the ARCC visit.

**Figure 5:**
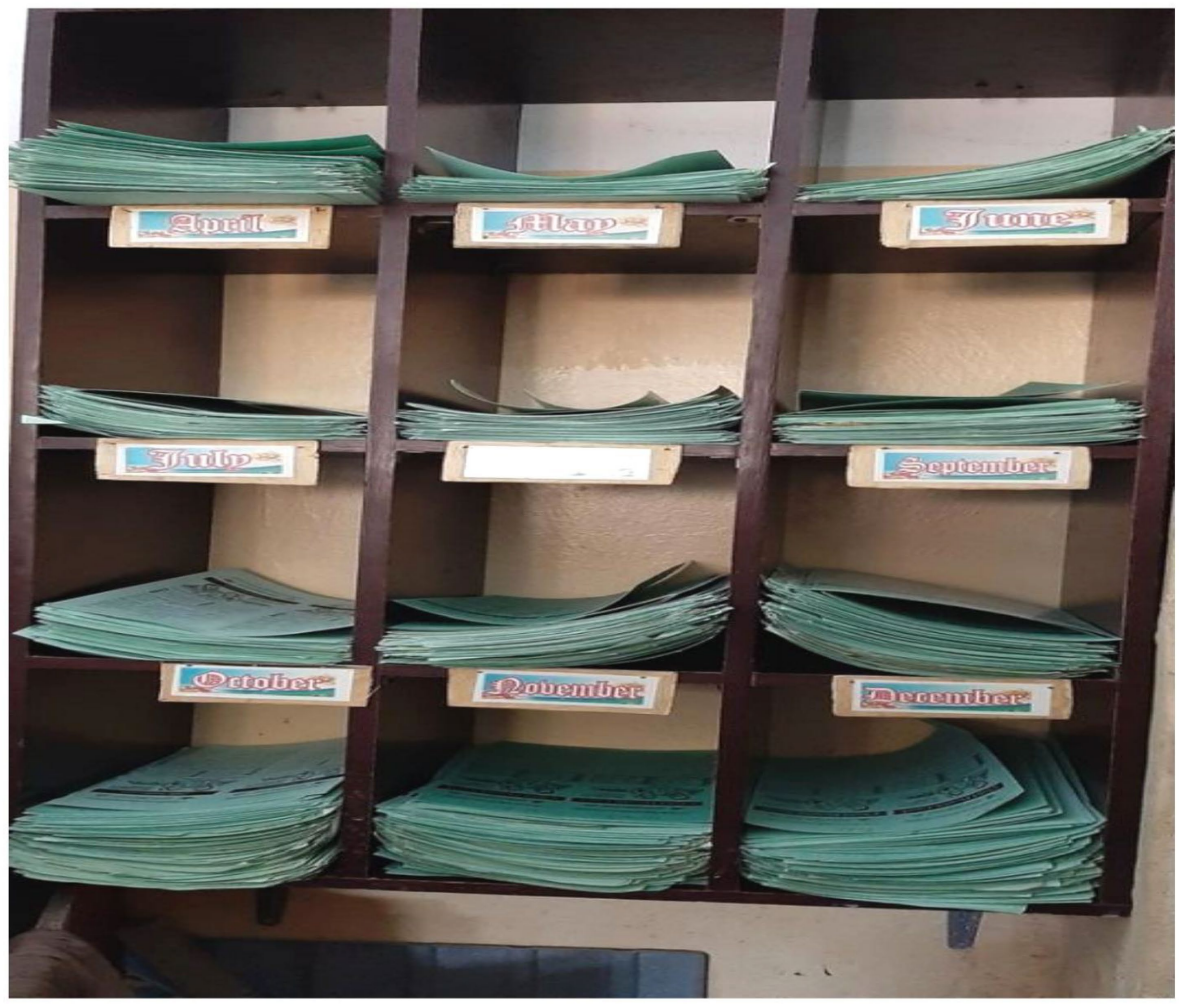
Client related and health records were well kept and reviewed during the visit.

See Figures 1–5 for some pictorials of methods during the field visit.

### Design and rationale

We adopted a narrative reporting design suited to integrating heterogeneous evidence—quantitative indicators, qualitative site-visit observations, policy documents, and operational lessons—from 2008 to 2020. The aim was synthesis and interpretation rather than meta-analysis.

### Data sources and selection

Sources included: (a) ARCC verification guidelines and Nigeria field-visit reports; (b) NPHCDA programme records (microplans, cold-chain temperature logs, SIA independent monitoring, and EOC daily situation reports); (c) WHO/UNICEF documentation on AFP and environmental surveillance, outbreak response, and RI strengthening; and (d) peer-reviewed publications on polio epidemiology, vaccine acceptance, and programme impact in Nigeria and analogous settings. Documents were screened for relevance to Kano and the ARCC verification questions. Materials unrelated to polio, lacking empirical content, or outside the time window were excluded.

### Kano health-system context

Kano is among Nigeria’s most populous states, with ~14 million residents across 44 Local Government Areas (LGAs); approximately 45% are children under 15 years—the core denominator for AFP surveillance. The service platform comprises >1,500 primary health-care facilities, several secondary hospitals, and tertiary referral centres. RI is delivered through fixed, outreach, and mobile sessions. The surveillance network includes Disease Surveillance and Notification Officers (DSNOs), community informants, laboratories linked to the Global Polio Laboratory Network, and increasingly, environmental sampling points in high-risk catchments (6, 8, 11–14).

### Site selection and visits

**Figure 6:**
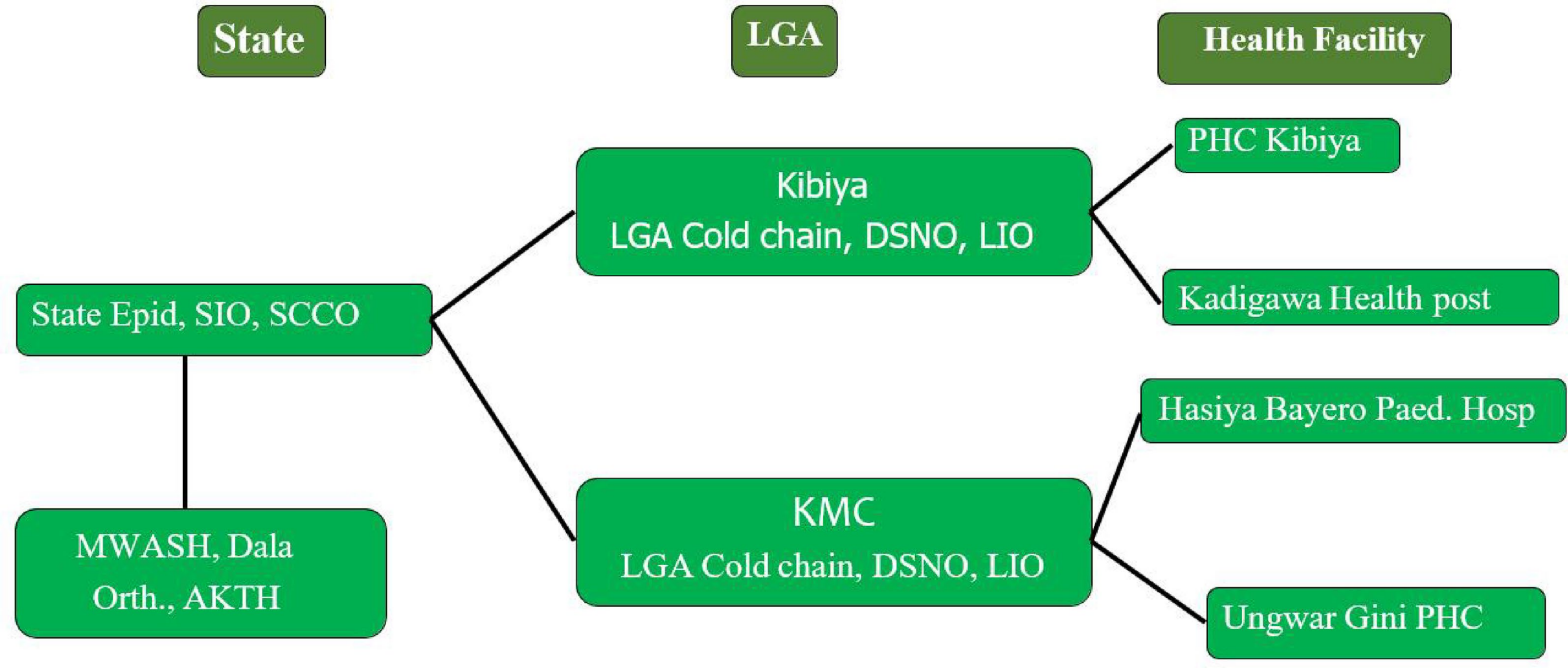
Verification framework and variables.

Two local government areas—Kano Municipal and Kibiya (LGAs), and two health facilities each in the selected LGAs were visited to review documentation across the WHO-recommended pillars of polio eradication. Similar to the state-level visits, the LGA immunisation office, Cold chain office, and the disease surveillance notification officers in these respective LGAs were visited for the review of records and reporting forms. The LGAs were selected based on the urban–rural characterisation, polio eradication indicators such as routine and supplemental immunisation coverages, and AFP surveillance indicators performance. These indicators were also considered in the selection of health facilities for the Team’s visit in the selected LGAs with key activities conducted and findings represented in the subsequent sections (Figure 6).

The plate below shows the schematic representation of site visit in the LGAs,

We abstracted AFP indicators (non-polio AFP rate per 100,000 children <15 years; stool adequacy), environmental surveillance coverage (sites, frequency, positivity), SIA quality measures (Lot Quality Assurance Sampling, independent monitoring), RI coverage (e.g., pentavalent-3/DTP3), documentation quality (microplans, CIFs, line lists, outbreak reports), and evidence of governance/coordination (EOC records). Qualitative variables included leadership engagement, community mobilisation strategies, and the use of digital tools—Auto-Visual AFP Ditection and Reporting (AVADAR) for case notification and GIS for microplanning and coverage validation.

### Quality considerations

Given the programmatic nature of many sources, we emphasised triangulation: comparing indicators across reports, cross-checking facility registers against LGA summaries, and reconciling independent monitoring with administrative coverage. ARCC peer-review notes and corrective-action logs were used to establish how identified gaps were addressed during the verification cycle.

## Results

### Surveillance performance and sensitivity

Kano’s non-polio AFP (NPAFP) rate exceeded the WHO threshold (≥2/100,000 children <15 years) in most LGAs in the years preceding certification. Where shortfalls were detected, they triggered targeted active-case searches, supportive supervision, and expansion of community informants. Stool adequacy generally met or approached the ≥80% benchmark, reflecting improved specimen collection and transport discipline. Environmental surveillance, expanded to strategically selected urban drains and high-risk catchments, provided an additional safety net for silent transmission; where polioviruses were detected historically, intensified mop-ups and microplan revisions followed.

AVADAR improved the timeliness and completeness of suspected AFP reporting. Community informants—teachers, religious leaders, patent-medicine vendors—received periodic prompts via the application to identify and report cases, reducing missed or late notifications.

DSNOs used dashboards to prioritise site visits, and feedback loops shortened the interval from notification to investigation.

### Routine immunisation and SIAs

#### Timeline of key milestones and quantitative signals 2008–2010

High transmission period with repeated SIAs and the early roll-out of independent monitoring. Microplanning at ward level was largely paper-based; settlement lists were incomplete and often missed nomadic clusters. Early corrective actions focused on mapping transit points, intensifying evening and market-day vaccination, and introducing incentives for caregivers (e.g., soap, sachet salt) to counter fatigue. AFP surveillance relied predominantly on facility-based reports; informal networks of informants were *ad hoc* (13–16).

Between 2010 and 2019, RI indicators trended upward. Administrative and independent-monitoring evidence pointed to gains in Penta-3/DTP3 coverage in the majority of LGAs, driven by better microplanning, session scheduling, and cold-chain reliability. Integration with maternal, newborn, and child-health (MNCH) services at fixed posts increased caregiver convenience. SIAs were conducted at a high cadence, with Lot Quality Assurance Sampling (LQAS) used to detect pockets of under-performance; corrective actions included redeploying teams, increasing transit vaccination, and engaging influencers in wards with persistent refusals.

Between 2009 and 2017 there were 61 polio SIAs in Kano. 5–6 rounds of SIAs are held yearly. As a result of the COVID 19 pandemic of 202 SIAs and routine immunisation witnessed a setback. However after the ARCC visit of that same year there was an improvement. From 8.5% RI coverage (Penta 3) in 2017 the figure went up to 47% in 2019 and then 77% in 2021 (17).

In August 2021, in response to cVDPV2 outbreak in Kano and Shortly after the ARCC visit 3,297,347 children aged 0–59 months (representing 99% of target) were vaccinated in the nOPV2 (novel oral polio vaccine type 2) campaign (18, 19). SIA rounds and outbreak responses are carried out 6–8 times a year particularly in identified high risk areas.

From this upward tragectory the state has remained free of wild polio transmission. However pockets of undervaccinated children still abound in only two high-risk local governments of Sumaila and Kano Municipal Area Councis.

### Documentation quality and data integrity

Verification placed heavy emphasis on documentation. Facility registers, CIFs, outbreak investigation files, and microplans were reviewed across high- and low-performing LGAs. Gaps—e.g., incomplete CIFs, absent neurologist assessments for residual paralysis, and discontinuities in temperature-log charts—were promptly corrected through mentoring and job-aids. GIS microplans helped validate settlement lists and ensured that household denominators aligned with service plans. EOC daily sitreps recorded actions taken and tracked follow-through, creating an auditable trail.

### Governance, coordination, and community engagement

Political leadership, including visible involvement of state executives and LGA chairs, bolstered partner alignment and accountability. The EOC structure enabled rapid decision-making and a culture of data use. Social mobilisation drew on the credibility of traditional rulers and Muslim clerics who publicly endorsed OPV after sustained dialogue and risk communication. Community-based organisations and women’s groups supported door-to-door counselling, while integrated health camps (vitamin A, deworming, malaria nets) created perceived value for caregivers beyond polio drops alone.

### Capacity building and cross-learning

Continuous training for DSNOs, RI focal persons, vaccinators, and data clerks addressed staff turnover and skill gaps. Peer-review exchanges with other states facilitated diffusion of good practices. As verification progressed, Kano teams increasingly contributed mentors to neighbouring states, reinforcing national coherence.

### Risk mitigation for cVDPV and importations

The programme instituted risk-based surveillance intensification in border LGAs and transit hubs and maintained readiness for rapid outbreak response. Communication materials were updated to explain cVDPV risks and the rationale for continued vaccination despite the absence of Wild Polio Virus (WPV), mitigating complacency (11–13, 20).

## Discussion

The Nigerian polio certification process followed the process for global polio certification (19). Members of the National Certification Committee comprised of clinicians and other public heath experts assigned by the National Polio Emergency Operations Centre, and the Ministry of Health as described by Datta et al. (18). The process adopted by the National Certification Committee in Kano which highlighted advocacy to the state institutions, visitation of identified sites to assess the structure to detect threats of polio virus outbreaks and advancing Global Polio Eradication Initiatives (GPEI) priorities which included interruption of vaccine-derived polio virus (vDPV) transmission all align with global practices for polio eradication (18, 19).

Kano state demonstrated resilience and determination in ensuring that the total number of cases remained at zero, and optimum immunization coverages are maintained through routine and supplemental vaccination exercises. The adoption of local strategies including the use of religious and traditional institutions reflects a classic demonstration about the need to integrate local contexts in addressing wicked public health problems (21). Community structures in Kano which included health and non-health actors led the resolution of pockets vaccine hesitancy across the state. These local entities initiated the translation of polio education materials into indigenous Hausa language to further drive readability and comprehension for non-English speakers, which contributed to vaccine acceptance, a major milestone in interrupting wild polio virus transmission.

### Implications for other programmes

Eradication efforts for measles or yellow fever can borrow several design elements: risk-based microplanning using GIS; embedded community informant networks; EOC-style coordination; and integrated service packages to increase perceived value. However, transfer must be context-sensitive: authority structures, mobility patterns, and media ecosystems vary. Pilot-test, learn, and iterate before scaling (6, 8, 11–13, 15–17, 22, 23) (13).

### Limitations

This report synthesises programmatic and published evidence but does not conduct primary data collection. The reliance on administrative reports and grey literature introduces risks of reporting bias and incomplete denominator accuracy. While ARCC verification offers an external check, some indicators—particularly RI coverage—are known to vary by source (administrative vs. survey). Finally, the experience is specific to Kano’s social and political context; extrapolation should be accompanied by contextual appraisal (6, 8).

## Conclusion and recommendations

Kano’s verification journey demonstrates that eradication is a systems achievement: leadership that insists on data use; communities that are partners rather than passive recipients; and platforms that turn information into timely action. To sustain gains and generalise lessons, we recommend: (1) institutionalise EOCs for all-hazards preparedness; (2) maintain high-quality AFP and environmental surveillance with periodic external reviews; (3) invest in RI quality, especially cold-chain reliability and session scheduling; (4) preserve and expand community partnerships with clerics, traditional rulers, and women’s groups; (5) mainstream digital tools (AVADAR, GIS) into routine supervisory workflows; and (6) plan and budget for transition of polio assets to PHC priorities. These steps will reduce the risk of cVDPV emergence and ensure that the infrastructure built for polio continues to serve broader public-health goals (11–13).

Kano State’s journey from a persistent polio transmission zone to a polio-free region illustrates the power of integrated public health strategies. Surveillance innovations like AVADAR, strong community engagement, rigorous monitoring, and multi-level collaboration were pivotal. The role of ARCC in validating and certifying these efforts underscores the importance of independent verification in public health milestones. Sustaining polio-free status will require continued vigilance, addressing cVDPV threats, and leveraging the polio infrastructure for broader health system strengthening (6, 8).

## References

[1] Fomban Leke RG King A Pallansch MA Tangermann RH Mkanda P. Certifying the interruption of wild poliovirus transmission in the WHO African region on the turbulent journey to a polio-free world. Lancet Glob Health. (2020) 8:e1345–51. doi: 10.1016/S2214-109X(20)30382-X 32916086 PMC7525084

[2] Nasir UN Bandyopadhyay AS Montagnani F Akite JE Mungu EB Uche IV. Polio elimination in Nigeria: a review. Hum Vaccin Immunother. (2016) 12:658–63. doi: 10.1080/21645515.2015.1088617, PMID: 26383769 PMC4964709

[3] Verma H Iliyasu Z Craig KT Molodecky NA Urua U Jibir BW. Trends in poliovirus seroprevalence in Kano state, northern Nigeria. Clin Infect Dis. (2018) 67:S103–9. doi: 10.1093/cid/ciy637, PMID: 30376090 PMC6206109

[4] Ahmed SH Nguku P Gidado SO Bawa MK Shehu UL Abdullahi A. Progress toward poliomyelitis eradication in Kano state, Nigeria, 2010–2017. Pan Afr Med J. (2021) 40:9. doi: 10.11604/pamj.supp.2021.40.1.19318 PMC9474848 36157557

[5] Effiong F Ladan D Oyebanji O. Nigeria’s polio elimination playbook: lessons to strengthen other outbreak responses’. J Global Biosecurity. (2021) 3:127. doi: 10.31646/gbio.127

[6] Global Polio Eradication Initiative (GPEI) (2022) ‘Polio eradication strategy 2022–2026: delivering on a promise (Executive Summary)’. Available online at: https://polioeradication.org/wp-content/uploads/2021/06/polio-eradication-new-Strategy-2022-26-Executive-Summary.pdf (Accessed: 19 September 2025)

[7] Wakabi W. Opponents stymie fight against polio in Nigeria. Can Med Assoc J. (2008) 179:891. doi: 10.1503/cmaj.081416, PMID: 18936452 PMC2565712

[8] World Health Organization Regional Office for Africa (WHO AFRO) (2020) ‘Africa eradicates wild poliovirus’. Available online at: https://www.afro.who.int/news/africa-eradicates-wild-poliovirus (Accessed: 19 September 2025)

[9] Nasir S-G Aliyu G Ya’u I. From intense rejection to advocacy: how Muslim clerics were engaged in a polio eradication initiative in northern Nigeria. PLoS Med. (2014) 11:e1001687. doi: 10.1371/journal.pmed.1001687 25093661 PMC4122353

[10] Centers for Disease Control and Prevention (CDC). Progress toward poliomyelitis eradication—Nigeria, January 2018–May 2019. Morb Mortal Wkly Rep. (2019) 68:642–6. 10.15585/mmwr.mm6829a3 PMC6660103 31344023

[11] Braka F Michael A Joseph B. The role of polio emergency operations centers (EOCs) in strengthening public health response. Oxford Open Infectious Dis. (2023) 3:ofad026. doi: 10.1093/ooid/ofad026

[12] Erbeto T Obasi I Eze N. Providing information for decision-making in the Nigerian National Emergency Operations Center: the data working group experience. Oxford Open Infectious Dis. (2023) 3:ofad025. doi: 10.1093/ooid/ofad025

[13] GRID3. (2021). ‘GRID3 microplanning maps to support immunisation activities in Nigeria’. Available online at: https://grid3.org/category/countries (Accessed on 19 September 2025)

[14] Hamisu AW Adamu US Craig KT. Characterizing environmental surveillance sites in Nigeria, 2018–2019. J Infect Dis. (2020) 221:S291–300. doi: 10.1093/infdis/jiaa168

[15] Diallo M Goto A Dia AT Sanogo K Minta D Kone S. ‘Auto-visual AFP detection and reporting (AVADAR): a digital tool for strengthening acute flaccid paralysis surveillance’, health. Security. (2021) 19:S47–55. doi: 10.1089/hs.2020.0160

[16] Ticha JM Okeibunor JC Ntaji MI Itiola AJ Banda R. Outcomes of the deployment of the auto-visual acute flaccid paralysis detection and reporting (AVADAR) system for improved AFP detection in the Lake Chad region. JMIR Public Health Surveill. (2020) 6:e18950. doi: 10.2196/18950 33263550 PMC7744265

[17] Global Advisory Committee on Vaccine Safety (GACVS), WHO (2023) ‘Poliovirus vaccines: nOPV2 use and prequalification update’. Available online at: https://www.who.int/groups/global-advisory-committee-on-vaccine-safety/topics/poliovirus-vaccines (Accessed: 19 September 2025)

[18] Datta SD Tangermann RH Reef S Schluter W Adams A. National, regional and global certification bodies for polio eradication: a framework for verifying measles elimination. J Infect Dis. (2017) 216:S351–4. doi: 10.1093/infdis/jiw578 28838172 PMC5853984

[19] Smith J Leke R Adams A Tangermann RH. Certification of polio eradication: process and lessons learned. Bull World Health Organ. (2004) 82:24–30. doi: 10.1590/S0042-96862004000100007 15106297 PMC2585869

[20] Polio Eradication (GPEI) – Nigeria Country Page (2023) ‘Nigeria: progress and updates’. Available online at: https://www.archive.polioeradication.org/countries/nigeria/ (Accessed: 19 September 2025)

[21] Shakeel SI Brown M Sethi S Mackey TK. Achieving the end game: employing “vaccine diplomacy” to eradicate polio in Pakistan. BMC Public Health. (2019) 19:79. doi: 10.1186/s12889-019-6393-1, PMID: 30654797 PMC6337835

[22] Asekun A Braka F Fatiregun A. Deployment of novel oral polio vaccine type 2 under WHO emergency use listing to curb outbreaks of circulating vaccine-derived poliovirus type 2—Nigeria, 2020–2021. Oxford Open Infectious Dis. (2023) 3:ofad027. doi: 10.1093/ooid/ofad027

[23] Voorman A Assiri A Bandyopadhyay AS. Impact of supplementary immunization activities using novel OPV2 on cVDPV2 outbreaks in Nigeria. J Infect Dis. (2024) 229:805–14. doi: 10.1093/infdis/jiad401 37357964 PMC10938209

